# Investigating the Obsessive and Compulsive Features of Cyberchondria: A Holistic Review

**DOI:** 10.3389/fpsyg.2022.897426

**Published:** 2022-07-04

**Authors:** Yi Yang, Na Ta, Zhanghao Li

**Affiliations:** ^1^School of Chinese Culture and Communication, Beijing International Studies University, Beijing, China; ^2^School of Journalism and Communication, Renmin University of China, Beijing, China; ^3^Computational Communication Research Center, Beijing Normal University, Zhuhai, China; ^4^School of Journalism and Communication, Beijing Normal University, Beijing, China

**Keywords:** cyberchondria, metacognition, unverified information, biased information environment, obsessive and compulsive searching

## Abstract

**Background:**

Cyberchondria has been brought into sharp focus during the COVID-19 health emergency; it refers to individuals who obsessively and compulsively search for health information online, resulting in excessive health concerns. Recent scholarship focuses on its obsessive and compulsive aspect, following a biopsychosocial approach as opposed to a pathology of health anxiety. It lacks interpretation of the socio-psychological dynamics between the dimensions.

**Objective:**

This review aims to propose a holistic view toward understanding cyberchondria as an obsessive–compulsive syndrome and considers possible interventions. It specifically seeks to explain cyberchondria from diversified mediator variables and to pinpoint connections between each perspective.

**Methodology:**

Comprehensive searches of databases such as *PubMed* and *Springer* were conducted to identify English articles relating to cyberchondria from 2001 to 2022. Based on a systematic filtering process, 27 articles were finally reviewed.

**Findings:**

The authors compare and confirm three forecasts to predict cyberchondria, associating it with individual metacognition, uncertainty of unverified information, and algorithm-driven, biased information environments.

**Value:**

Theoretically, a holistic framework is proposed to explain the obsessive and compulsive features of cyberchondria. Clinically, the research calls for more professional psychoeducation and chain screening of cyberchondria and other psychological disorders. Socially, it promotes support for risk-sensitive, information-deficient groups during pandemics like COVID-19. It also stresses more careful use of algorithm-driven search engine technology for platforms delivering medical information. Future research may explore areas such as the association between cyberchondria and other social-related disorders, as well as correlations among cyberchondria, obsessive and compulsive disorders, medical trust, and algorithm-driven search results.

## Introduction

**Figure 1:**
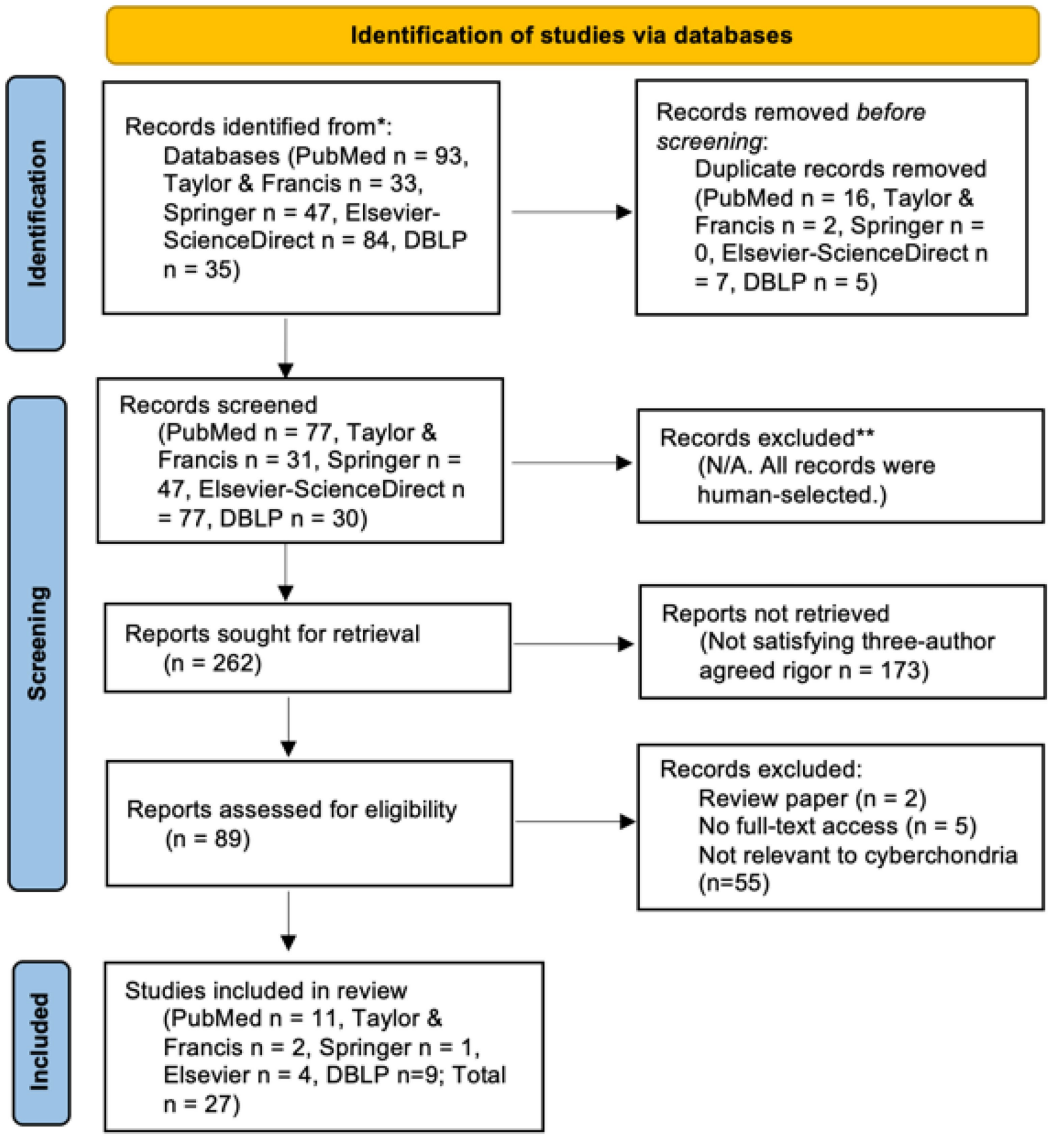
The PRISMA flow diagram of the studies included in this review.

The COVID-19 pandemic and its numerous and varied symptomologies have spawned an epidemic of online symptom-checking, resulting in a crisis of psychological wellbeing within the public and spotlighting the condition of cyberchondria. Cyberchondria features compulsive online researching of health-related symptoms (Fergus and Spada, 2017; Newby and McElroy, 2020). Existing studies, under the COVID-19 pandemics, interpret cyberchondria with three different models (Figure 1). In the pathology model, cyberchondria is treated as a sub-symptom of health anxiety. Studies demonstrate that health anxiety is positively associated with online health information seeking (OHIS), resulting in cyberchondria (Mathes et al., 2018; McMullan et al., 2019). Conversely, the other two models explain cyberchondria within the obsessive–compulsive disorder (OCD) framework. They also consider health anxiety but place more importance on the repeated online health-related searches and their antecedents. In the hybrid model, the condition is understood as an escalating distress that eventually develops into cyberchondria—generated due to uncontrollable worries about illness and excessive and compulsive searching behaviors (Brown et al., 2019). Significantly, in the integrative model, Zheng et al. (2021) proved that negative metacognitive beliefs could function as a boundary condition for regular OHIS, resulting in cyberchondria.

Our review also focus on the obsessive and compulsive dimension of cyberchondria rather than regarding it as a single pathology; however, our review differs from the hybrid and integrative models. The former centers on how health beliefs might impact OHIS but fails to analyze the development dynamics of cyberchondria. The latter follows a behaviorism approach, emphasizing a concrete process during which online-seeking behaviors generate effects; however, relevant studies lack in analyses of the interconnections between each antecedent and its associations with cyberchondria. The following OCD studies on cyberchondria inspire us to propose a more holistic view. Zheng et al. (2020) identify psychological risk factors, information factors, and personality traits associated with cyberchondria. Others suggest a biopsychosocial approach. Physically, a threatening interpretation of bodily sensations or benign symptoms might increase bodily sensations—such as increased heart rate or sweating—during online searches, and develop into cyberchondria (Witthöft and Hiller, 2010). Psychologically, individuals with anxiety traits are more likely to become anxious about viruses, leading to cyberchondria (Jungmann and Witthöft, 2020). Socially, users’ online exposures to threatening medical information, regardless of whether they had health anxiety before OHIS, could increase cyberchondria (Vismara et al., 2020). Drawn on these studies, our review attempts to explain the interconnected relationships between the cognitive, social, and technical antecedents.

Understanding cyberchondria under an OCD framework also highlights its public health significance in this digital age with a populace suffering from pandemics. It emphasizes excessive and repeated online health-related searches, shedding light on the paradoxical role of the internet during this period of long-distance socialization, quarantine, and isolation. Wu et al. (2021) illustrate how repeated online media consumption during the pandemic amplifies individual anxiety and fear, leading to cyberchondria. The internet is unquestionably an information resource, but it also contributes to public anxiety and distrust of unverified information, as well as vulnerabilities perceived by users due to biased search results shaped by algorithm-driven technologies. As such, this review will also provide novel analyses on how the emerging algorithm-driven search engine technologies shape biased information environments that impact cyberchondria.

## Question and Objective

This targeted review^1^ closely examines one question: Is there a holistic view that explains cyberchondria as an OCD beyond a single pathology?

Based on peer-reviewed published articles in this field, this work aims to compare different research foci to build an informative analysis of literature on the obsessive and compulsive feature of cyberchondria. It specifically endeavors to clearly highlight the main results that emerged in each area, as well as to pinpoint potential connections between them—ways in which they could be combined to advance the understanding of cyberchondria and the possible interventions.

## Method and Design

The authors conducted a comprehensive search of five databases consisting of health-related studies across multiple disciplines: one medical science databases (PubMed), four comprehensive databases (Taylor & Francis, Springer, Elsevier) and one information technology database (DBLP).

The search term “cyberchondria”^2^ was applied to titles, abstracts, and keyword fields to filter out irrelevant studies. The screening criteria were peer-reviewed English articles published that regard cyberchondria as an OCD rather than a sub-symptom of health anxiety. Reference lists of the included papers were also checked for inclusiveness. Figure 1 shows the PRISMA flowchart of the literature screening process.

Three authors examined these articles separately and agreed on the final sample of 27 published from 2001 to 2022, from which the following information was extracted: source, database, method, and main findings. The authors also categorized samples and summarized three perspectives: cognitive, social, and technological. All information is identified in Table 1.

**Table 1:** The final sample of literature reviewed in this article.

Source	Database	Citation	IF	Method	Keyword	Main findings	Perspective
Bailey and Wells, 2015*	PubMed	42	3.222	Survey	Metacognition, health anxiety, exploratory factor analysis, confirmatory factor analysis, and validity	It reports on the development and initial evaluation of a new specific metacognitive measure of health anxiety, the Metacognitions Questionnaire-Health Anxiety (MCQ-HA).	Cognitive
Bajcar and Babiak, 2020	Elsevier	9	3.004	Survey	Cyberchondria, neuroticism, intolerance of uncertainty, defensive pessimism, and mediation model	Results have revealed that, of the FFM personality traits, only neuroticism was associated with cyberchondria. The effect of neuroticism on cyberchondria was confirmed	
Fergus and Spada, 2017	PubMed	77	2.938	Survey	Anxiety sensitivity, cyberchondria, intolerance of uncertainty, metacognition, metacognitive beliefs, and problematic internet use	Cyberchondria shared a moderate to strong association with problematic Internet use and metacognitive beliefs.	
Fergus and Spada, 2018*	PubMed	38	5.264	Survey	Cyberchondria, beliefs about rituals, health anxiety, metacognitive beliefs, and stop signals	Beliefs about rituals and stop signals emerged as relatively specific to cyberchondria versus health anxiety, which preliminary support for a metacognitive conceptualization of cyberchondria	
Jungmann and Witthöft, 2020	Elsevier	279	5.264	Survey	Cyberchondria, COVID-19, emotion regulation, health anxiety, and virus anxiety	Cyberchondria Pandemic showed positive correlations with current virus anxiety, and this relationship was additionally moderated by trait health anxiety. A negative correlation was found between the perception of being informed about the pandemic and the current virus anxiety.	
Oniszczenko, 2021*	PubMed	4	3.240	Survey	-	Cyberchondria positively correlated with both COVID-19 fears scales, though the correlation coefficients were medium. Based on the results of linear regression analysis, only anxious temperament and COVID-19 fear of self-infection were significant predictors of cyberchondria.	
Sofia et al., 2022	PubMed	0	2.885	Survey	Cyberchondria, health anxiety, health cognitions, and metacognitions about health anxiety	Metacognition about health anxiety relating to beliefs about the uncontrollability of thoughts was the only significant predictor of prospective cyberchondria scores when controlling for health anxiety.	
Zheng et al., 2021	Elsevier	6	6.182	Survey	Cyberchondria, information insufficiency, health anxiety, online health information seeking, and negative metacognitive beliefs	This study further identifies negative metacognitive beliefs as a boundary condition for how regular OHIS results in cyberchondria.	
Allen et al., 2014	Springer	164	4.344	Mixed method	Inter-organizational information systems, information sharing, activity theory, and emergency response	Online sharing and communication have a proven positive on the recovery of individual in crisis.	Social
Bora et al., 2018	Taylor & Francis	107	3.802	Content analysis	Public health, internet, social media, health communication, health information, and pandemic	Misinformation will be spread more quickly than information during a public health event and further threaten people’s mental health.		
Cinelli et al., 2020	PubMed	1,125	4.13	Data-driven	-	Information spreading is driven by the interaction paradigm imposed by the specific social media or/and by the specific interaction patterns of groups of users engaged with the topic.		
Maftei and Holman, 2020*	Taylor & Francis	21	2.990	Survey	Cyberchondria, coronavirus, neuroticism, optimism, and age	Among elderly participants, the psychologically protective influence of optimism against cyberchondria emerged as larger than the opposite effect of neuroticism.		
McElroy et al., 2019*	PubMed	10	4.157	Survey	Cyberchondria, health anxiety, self-diagnosis, and general anxiety	Cyberchondria Severity Scale (CSS 12) highlighted four indicators, coercion, suffering, excess, seeking comfort.		
Parsons and Alden, 2022*	Elsevier	0	1.418	Survey	Obsessive–compulsive disorder, reassurance-seeking, shame, fear of self, and cyberchondria	This research identifies symptoms and characteristics that may be linked to more frequent online reassurance-seeking in particular. Unacceptable thoughts appear uniquely related to reassurance-seeking from non-interactive online sources.		
Patwary et al., 2021	PubMed	1	3.044	Survey	Information source trust, coronavirus, SARS-CoV-2, mental health, COVID-19 stressor, and Global south	Trusting social media to provide accurate COVID-19 information may exacerbate poor mental health, while trusting traditional media (i.e., television, radio, and the newspaper) may have stress-buffering effects.		
Rovetta and Bhagavathula, 2020	PubMed	88	5.43	Data-driven	COVID-19, coronavirus, Google, Instagram, infodemiology, infodemic, and social media	Globally, there is a growing interest in COVID-19, and numerous infodemic monikers continue to circulate on the Internet.		
Starcevic et al., 2020a	PubMed	38	N.A.	Theoretical research	COVID-19, cyberchondria, information overload, intolerance of uncertainty, online health information, online health information literacy, online health searching, public health, reassurance seeking, and uncertainty	This model of cyberchondria during the COVID19 pandemic contributes to the literature by helping to understand the hypothesized rise in cyberchondria during public health emergencies and formulate a framework for prevention of cyberchondria and effective responding to it.	
Starcevic et al., 2020b	PubMed	35	5.285	Theoretical research	Cyberchondria, online health research, reassurance seeking, health anxiety, problematic internet use, and compulsivity	Most definitions of cyberchondria emphasize online health research associated with heightened distress or anxiety. The two theoretical models of cyberchondria involve reassurance seeking and specific metacognitive beliefs.	
Azzopardi et al., 2018*	DBLP	36	8.740	Data-driven	-	Search engines prioritize ranking over relevance to generate SERP, to improve the click through rate. Serious and negative health information in SERP have increased click through rate due to their high ranking.	Technological
Gao and Shah, 2020*	DBLP	46	6.222	Data-driven	Information retrieval, search engine bias, fairness ranking, relevance, diversity, and novelty	The ranking of the results in Search Engine Result Pages (SERP), especially by top-level search engines, is unbalanced and does not conform with the general diversity distribution.	
Gezici et al., 2021*	DBLP	3	2.293	Data-driven	Bias evaluation, fair ranking, search bias, and web search	Search engines are not necessarily neutral. Different search engines may have different ideological biases and present different search results to users.	
Joachims et al., 2017	DBLP	75	8.740	Data-driven	Implicit feedback, eyetracking, WWW search, and clickthrough	Internet users tend to click on top-ranking results, and are more likely to trust them, thus maintaining the ranking of these websites.	
Muussesa et al., 2014	DBLP	108	6.829	Survey	Compulsive internet use, psychological wellbeing, happiness, depression, and loneliness	Compulsive Internet Use (CIU) predicted increases in depression, loneliness and stress over time, and a decrease in happiness. No effect of CIU on the change in self-esteem was found. Further, happiness predicted a decrease in CIU over time.	
Schoenherr and White, 2014	DBLP	5	8.740	Data-driven	Health search, medical search, diagnosis, log/behavioral analysis, and cyberchondria	Based on exposure to online content, people may develop undue health concerns, believing that common and benign symptoms are explained by serious illnesses.	
Schultheiß and Lewandowski, 2021*	DBLP	2	3.282	Online survey	Information literacy, online survey, search engines, and user trust	Users strongly trust Google, yet they are unable to adequately evaluate its search results. Users with little search engine knowledge are more likely to trust and use Google than users with more knowledge.	
Virginia et al., 2021	DBLP	0	N.A.	Data-driven	Application, information retrieval, reranking, and search engine	Search engine algorithms attach importance to the relationship between user click behavior and result ordering. Internet users tend to click on top-ranking results.	
White and Horvitz, 2013	DBLP	19	2.043	Data-driven	Captions, biases, diagnostic search, and cyberchondria	Users are significantly more likely to examine and click on captions containing potentially-alarming medical terminology such as “heart attack” or “medical emergency” independent of result rank position.	

An asterisk (*) indicates that this article is of greater importance than others. A short line (-) indicates there are no keyword in this article.

Based on the literature review, the authors further compared three forecasts to predict cyberchondria.

### Forecast One

Cyberchondria is associated with problematic Internet use driven by an individual’s metacognition of the state of health.

### Forecast Two

Cyberchondria is associated with the escalating distress resulting from the unverifiable information produced when searching online information for reassurance.

### Forecast Three

Cyberchondria is associated with a biased information environment due to manipulated algorithm-driven search technologies.

## Results

### Quality Assessment of Articles Included^3^

Three authors conducted a comprehensive evaluation of the 27 included papers from seven aspects, including study design, the number of participants/groups, outcome measure, quality of intervention, suggesting possible intervention, quality of reporting, and generalizability. Seventeen papers were evaluated as of high quality in all aspects. Two papers did not carry out empirical research but provided theoretical-driven models to inspire further study on the obsessive and compulsive quality of cyberchondria. As many papers adopt method of questionnaire survey, some of them had a moderate performance in the choice of outcome measure and the quality of the intervention. See Table 2 for more details on the quality assessment.

**Table 2:** Quality assessment of article included.

Source	Method	Research design	Number of participants/groups	Choice of outcome measure	Quality of the intervention	Suggesting possible intervention	Quality of reporting	Generalisability	Perspective
Bailey and Wells, 2015	Survey	High	*N*_1_ = 351; *N*_2_ = 553; *N*_3_ = 259	High	High	Yes	High	High	Cognitive
Bajcar and Babiak, 2020	Survey	High	*N*_1_ = 381; *N*_2_ = 355	High	High	Yes	High	High	
Fergus and Spada, 2017	Survey	High	*N*_1_ = 337; *N*_2_ = 260	High	High	Yes	High	High	
Fergus and Spada, 2018	Survey	High	*N*_1_ = 330; *N*_2_ = 331	High	High	Yes	High	High	
Jungmann and Witthöft, 2020	Survey	High	*N* = 1,615	High	High	Yes	High	High	
Oniszczenko, 2021	Survey	High	*N* = 499	High	High	Yes	High	High	
Sofia Airoldi et al., 2022	Survey	High	*N* = 125	High	Moderate	Yes	High	High	
Zheng et al., 2021	Survey	High	*N* = 426	High	Moderate	Yes	High	High	
Allen et al., 2014	Mixed method	High	N.A.	Moderate	Moderate	Yes	High	High	Social
Bora et al., 2018	Content analysis	High	*N* = 101 videos	Moderate	High	Yes	High	High	
Cinelli et al., 2020	Data-driven	High	1,342,103 posts; 7,465,721 comments produced by 3,734,815 users	High	High	Yes	High	High	
Maftei and Holman, 2020	Survey	High	*N* = 880	High	High	Yes	High	High	
McElroy et al., 2019	Survey	High	*N* = 208	High	High	Yes	High	Moderate	
Parsons and Alden, 2022	Survey	High	*N* = 459	High	High	Yes	High	High	
Patwary et al., 2021	Survey	High	*N* = 744	High	High	Yes	High	High	
Rovetta and Bhagavathula, 2020	Data-driven	High	2,918,000 Hashtags	Moderate	High	Yes	High	High	
Starcevic et al., 2020a	Theoretical research	High	N.A.	N.A.	N.A.	Yes	High	High	
Starcevic et al., 2020b	Theoretical research	High	N.A.	N.A.	N.A.	Yes	High	High	
Azzopardi et al., 2018	Data-driven	High	More than 1,000 queries	High	High	Yes	High	High	Technological
Gao and Shah, 2020	Data-driven	High	100 Queries, and 100 results for each query	High	High	Yes	High	High	
Gezici et al., 2021	Data-driven	High	57 Query topics over two popular search engines	High	N. A.	Yes	High	High	
Joachims et al., 2017	Data-driven	High	10 Query tasks, 56 participants	High	High	Yes	High	Moderate	
Muussesa et al., 2014	Survey	High	Longitudinal study of 398 samples	High	High	No	High	High	
Schoenherr and White, 2014	Data-driven	High	More than 50,000 unique queries with 20,000 ~ 50,000 users	High	High	Yes	High	High	
Schultheiß and Lewandowski, 2021	Online survey	High	2012 Users	High	Moderate	No	High	Moderate	
Virginia et al., 2021	Data-driven	Moderate	1,386 Product data with 5 judges to label the data	Moderate	N.A.	No	High	Moderate	
White and Horvitz, 2013	Data-driven	High	8,732 Individuals number of queries 515 individuals to take a survey	High	High	Yes	High	High	

*Research design is based on three-authors-agreed appropriateness of study design to the research objective. Quality of intervention is based on whether the proposed intervention is evaluated to fit long-term treatment. Generalizability is based on whether the research conclusion fits repeated examinations in other contexts*.

**Figure 2:**
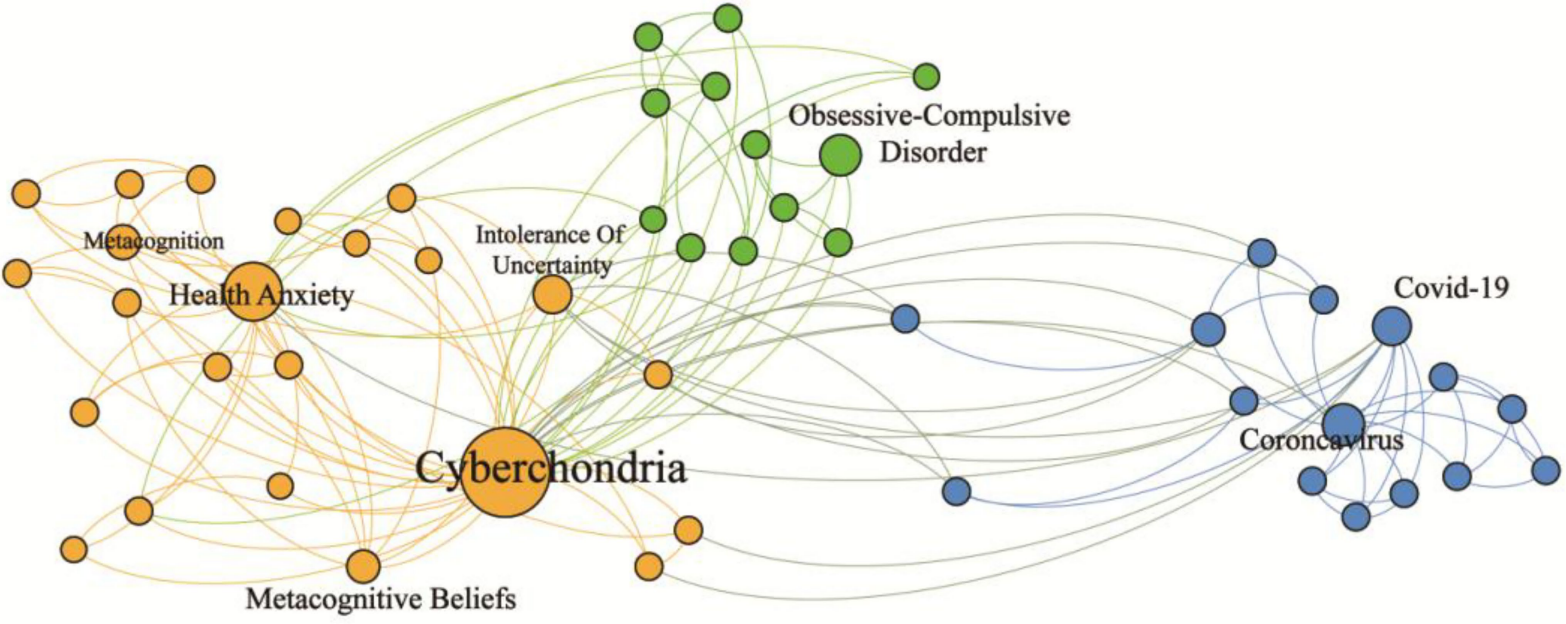
Co-occurrence network diagram of paper keywords related to cyberchondria. Figure shows a core subgroup of 50 nodes, whereby nodes represent keywords of articles and edges represent the connection between two keywords that appear in the same article. Data sources are from 27 included articles, including 84 nodes and 197 undirected edges. The size of a node represents the weight of the edge. Gephi, a network graph software, was used to map the relationships between these keywords. As shown in this figure, existing studies examine cyberchondria under its associations with the ongoing COVID-19 emergency. Most studies focus on health anxiety pathology. The rest focuses on obsessive–compulsive disorder, among which metacognition and intolerance of uncertainty are important concepts. This figure suggests research directions for the present study.

After reviewing all included articles, the authors compared and confirmed three perspectives for a holistic understanding of cyberchondria (Figure 2).

### Cognitive Perspective: Problematic Internet Use Driven by Metacognitions

Cognitive psychology literature links the obsessive and compulsive feature of cyberchondria when an individual’s problematic Internet use is driven by metacognition of their health state. As a belief chain that precedes health anxiety, metacognition indicates that an individuals’ pre-thoughts about their health state will significantly impact their physical health, emotions, and obsessive and compulsive behaviors when coping with cyberchondria. Metacognition is proven to be associated with psychological disturbance and then with cyberchondria (Bailey and Wells, 2015). Scholarship focus is on anxiety traits (Jungmann and Witthöft, 2020) and neuroticism (Oniszczenko and Stanisławiak, 2019). Those with anxiety traits are more likely to adopt a threatening perspective to understand benign symptoms or external risks; this introduces fear and anxiety that may result in obsessive and compulsive OHIS (Oniszczenko, 2021). Those with high intolerance of uncertainty (IU) tend to adopt a defensive pessimism (DP) strategy. This means that they usually think of all the things that could go wrong and then work diligently to prevent the perceived upcoming worst-case scenario (Norem and Chang, 2002). The DP is directly linked with obsessive and compulsive OHIS; therefore, it is more likely to cause cyberchondria (Bajcar and Babiak, 2020).

These studies echo different directions of metacognition proposed by Fergus and Spada (2018). Specifically, anxiety traits correspond to negative metacognition, i.e., “Uncontrolled thinking about illness can cause illness.” The condition develops into cyberchondria through mediation of anxiety and fear. The IU corresponds to positive metacognition that develops into cyberchondria through the mediation of self-protection, namely “Worrying about illness better equips us to defend ourselves against illness.” Others suggest that the effect of positive metacognition on cyberchondria is mediated by curiosity (Fergus and Spada, 2017). Ultimately, scholars pay more attention to the effect of negative metacognition on cyberchondria as an OCD (Airoldi et al., 2021; Zheng et al., 2021).

The cognitive perspective associates cyberchondria with obsessive and compulsive OHIS driven by metacognitions. However, following the biopsychosocial approach of the OCD framework, individual information exposure and social interaction warrants further examination. This will be discussed in the next section.

### Social Perspective: The Contradiction Between Information Needs and Information Verification

Socially focused studies have typically linked cyberchondria with the escalating psychological distress led by the tension between an individual’s reassurance-seeking needs and the uncertainty driven by unverified online information (Starcevic et al., 2020a). Those with OCD are more likely to have excessive reassurance-seeking (ERS); they rely on the Internet because seeking interpersonal reassurance may cause them shame and fear (Parsons and Alden, 2022). This trend has been amplified due to the health risks posed by COVID-19, especially since lockdowns and normalized social distancing have resulted in more Internet use to find information, socialize and fulfill emotional needs (Cinelli et al., 2020; Rovetta and Bhagavathula, 2020). However, this online information may not suffice, since OCD individuals need definitive explanations and trustworthy information sources (Starcevic et al., 2020b). Ironically, these internet users perceive their health as fragile and continue to search online, yet they cannot fully depend on the veracity of online information. Consequently, they continue searching and sharing, leading to cyberchondria (Bora et al., 2018; Patwary et al., 2021). Obsessive and compulsive OHIS positively correlates with an individual’s dependence on online information, information overload (White and Horvitz, 2009), uncertainty (Del Vicario et al., 2016), and perceived vulnerability (Laato et al., 2020). Therefore, cyberchondria occurs through the escalating psychological distress led by tension between information needs and the inability to verify information sources.

This perspective highlights the paradoxical interactions between humans and the Internet during health emergencies. Online sharing and communication have been proven to positively affect the recovery of individuals in crisis (Allen et al., 2014). A widely adopted intercultural cyberchondria severity scale (CSS-12; McElroy et al., 2019) highlights four indicators: coercion, suffering, excess, and seeking comfort. The last two reflect our social perspective. Maftei and Holman (2020) confirm that positive emotional support has helped prevent cyberchondria among the elderly during the COVID-19 pandemic. Their study shows the necessity of linking excessive and repeated OHIS with an individual’s need to seek reassurance.

Overall, individuals rely on online information but cannot verify it, resulting in exacerbation of cyberchondria. Yet, the social perspective often disregards technology as a powerful mediator of social significance; it should be viewed as an infrastructure for technological–social integration and not as simply a single external tool. This fascinating irony offers direction for further exploration.

### Technological Perspective: The Linkage Between Cyberchondria and Biased Information Environment

Regardless of whether health anxiety or OHIS is the first to occur, the information found in search results leads to an individual’s escalating anxiety (Starcevic, 2017). Understanding this information environment is key to examining cyberchondria under an OCD framework and predicting further interventions. Technologically focused studies associate cyberchondria with a biased online information environment driven by Search Engine Result Pages (SERP) algorithms.

Since titles of SERP results affect the clicking of corresponding web page results (White and Horvitz, 2013), users are more likely to view and click on titles containing potentially severe medical terms. Specifically, the introduction of certain words in search results might aggravate cyberchondria. Additionally, search engines automatically generate variants of query terms when composing SERP to improve performance, which in turn can develop into issues of luring clicks and reinforcing worries. However, based on such complex algorithms, SERP do not necessarily cover all topics related to query terms and may be biased toward specific views (Gezici et al., 2021). Individuals sometimes perceive benign symptoms as serious diseases after viewing online content and may experience psychological distress (Schoenherr and White, 2014).

Echoing the social perspective, users trust search engines but cannot fully evaluate the search results (Schultheiß and Lewandowski, 2021); this makes them vulnerable. The more trusting users are more likely to rely on the system and click on SERP; they are also more likely to classify common benign symptoms as serious diseases. Meanwhile, indulgence in SERP can be regarded as a form of compulsive internet use that lowers wellbeing by predicting increases in depression and stress over time; the resulting decrease in happiness may affect health (Muussesa et al., 2014).

Thus, the manipulation of users’ obsessions with and trust of search engines forms a biased information environment. Users’ anxiety levels may increase after escalating compulsive online searching for reassurance—potentially cultivating cyberchondria.

## Discussion and Conclusion

### Principal Findings

This article summarizes, contrasts, and confirms cognitive, social, and technological mediation perspectives to understand cyberchondria holistically. Specifically, the cognitive perspective links cyberchondria with obsessive and compulsive OHIS driven by metacognitions. The social perspective associates cyberchondria with escalating distress caused by the tension between ERS needs and information verification. The technology perspective highlights how manipulated algorithm-driven search engines shape biased information environments and affect cyberchondria. Results also pinpoint potential connections between the perspectives that are listed below.

### Discussion and Future Directions

An individual’s metacognition is not isolated from others with broader social backgrounds. There is rich scholarship on social factors behind one’s beliefs and thoughts, such as escape from social pressures (Turkle, 1995) and gender differences (Laato et al., 2020). Individual cognition should be linked to the social dynamics that underpin searches; this linkage might also be the key to solving the recursive relationship between health-related anxiety and cyberchondria. Irrespective of whether cyberchondria causes or is a result of health-related anxiety, the group characteristics and use patterns of an individual cannot be ignored. As highlighted by Fergus and Dolan (2014), cyberchondria in teenagers often manifests as addictions to online gaming and pornography. Their findings show how the cognitive and social perspectives are continually engaged. More research is anticipated on the interconnected relationships between the obsessive and compulsive feature of cyberchondria and other social-related disorders, such as social anxiety and online gaming disorder.

Furthermore, since modern social support systems are increasingly mediated online by socially mobile technologies, social and technological perspectives are inseparable. Although most studies treat search behaviors as central to the study of cyberchondria, we go further by recognizing that search results are driven by manipulated algorithms. Special attention is given to algorithm completions. In addition, certain groups are in particular need of online social support at different physiological stages of life and are more likely to suffer from OCD. Their experiences of cyberchondria may not all be accurately traced through quantitative log collection. Therefore, the qualitative study of the cyberchondria-related experiences of groups such as pregnant women and elderly patients also merits further discussion.

Finally, the influence of algorithm-driven search engines cannot be fully understood in the absence of users’ reactions. Therefore, the technology perspective has shown how the algorithm and intelligent search technology increasingly comprise what Peters (2015, p. 37) called the infrastructure of social interaction. Echoing this prediction, some studies have explored how self-diagnosis is influenced by people sharing symptoms in online health-topic communities (Wheatley et al., 2003; Giles and Newbold, 2013); others have exhorted doctors to give patients more authoritative sources of information (Conell et al., 2016) or to deliver mediations such as internet cognitive behavioral therapy (Newby and McElroy, 2020). Therefore, further research is required on the relationship between cyberchondria, online source verification, and social trust. For instance, how could the standard of SERP be established with fair and verified results to alleviate concerns related to obsessive and compulsive viewing for health-related queries? Would the presence of more doctors in online communities improve the quality of shared information, thereby reducing cyberchondria? Such questions are worthy of further examination.

### Contribution and Limitation

The contributions of this study are three-fold. Theoretically, it proposes a more holistic view engaging cognitive, social, and technological perspectives, paving the way for a systematic framework to understand cyberchondria under the OCD framework wherein the three dimensions are interconnected. Clinically, it calls for chain-screening cyberchondria, obsessive and compulsive disorder, and other maladaptive social anxiety symptoms such as social anxiety and depression, as well as more professional psychoeducation. Socially, it appeals for more social support for risk-sensitive groups with high OCD potential, especially given the anxieties and panic caused by pandemics such as COVID-19 and the need to cope with long-distance socialization and quarantines. It also calls for a more just information environment. Specifically, when using algorithm-driven search engine technology to deliver health-related information, digital platforms should focus on the real distribution of the illness instead of simply pursuing potential patients’ click rates.

Concerning limitations, this study defends a holistic view rather than a single diagnostic view of understanding cyberchondria. Therefore, the authors may omit some papers during literature screening, especially those papers discussing the social dynamics behind symptoms.

### Conclusion

This targeted review, based on 27 peer-reviewed published articles from five authoritative databases, investigates cyberchondria to understand and assess treatment for the obsessive and compulsive symptoms of the condition from cognitive, social, and technological perspectives. The authors compare and confirm three forecasts to predict cyberchondria, stating that it is associated with an individual’s metacognition, the tension between seeking reassurance from information and uncertainty caused by unverified online information, and algorithm-driven intelligent search engine technologies. These three perspectives are continually and dynamically engaged, despite their different foci, and theoretically and practically contribute to the current human knowledge on cyberchondria. Future research may explore areas such as the association between cyberchondria and other social-related psychological disorders, how cyberchondria correlates with social trust mediated by algorithm-driven search engines, and how medical professionals might provide OCD patients with reassurance and information verification—thus decreasing the incidence of cyberchondria.

## Author Contributions

YY and NT contributed to the conception and design of the study. ZL organized the statistical analysis. YY, NT, and ZL wrote the first draft of the manuscript. All authors worked on revisions of the manuscript, performed the additional analysis, wrote the sections of the manuscript, contributed to manuscript revision, read the manuscript, and approved the submitted version.

## Funding

This study was supported by the 2022 New Teaching Staff Scientific Research Initiative Plan of Beijing International Studies University (KYQH22A008) and the Chinese National Social Science Foundation (21BXW061).

## Conflict of Interest

The authors declare that the research was conducted in the absence of any commercial or financial relationships that could be construed as a potential conflict of interest.

## Publisher’s Note

All claims expressed in this article are solely those of the authors and do not necessarily represent those of their affiliated organizations, or those of the publisher, the editors and the reviewers. Any product that may be evaluated in this article, or claim that may be made by its manufacturer, is not guaranteed or endorsed by the publisher.

## Footnotes

^1^The authors refer to Huelin et al. (2015) to define a non-systematic targeted review.

^2^To improve the comprehensiveness of the literature screening of the third perspective, authors combined the term “search engine” with the original search term “cyberchondria.”

^3^The citation number is not applicable to those articles published in and after the year of 2021.
